# Cystoscopically Guided Repair of a Posterior Perforation of Augmented Bladder

**DOI:** 10.1089/cren.2018.0065

**Published:** 2019-03-18

**Authors:** Felicia Balzano, Humberto Villarreal, Thomas E. Novak

**Affiliations:** Department of Urology, San Antonio Military Medical Center, San Antonio, Texas.

**Keywords:** bladder perforation, bladder augmentation, urinary diversion, instrumentation, translational medicine

## Abstract

***Background:*** Augmentation cystoplasty for the management of neurogenic bladder is one of the mainstays of pediatric urology. This procedure has multiple well-known complications. The most dangerous of these complications is bladder perforation, which has a mortality rate of 23% to 25% in large part caused by delayed presentation and sepsis. This case report discusses a novel method for identifying the perforation using endourologic techniques to allow for easier repair.

***Case Presentation:*** A 24-year-old woman with a history of spina bifida s/p augmentation cystoplasty with appendicovesicostomy and rectus fascia bladder neck sling 5 years ago presented to the emergency department with a 2-day history of decreased oral intake, nausea, vomiting, fevers, diffuse abdominal pain, and distention. She was found on CT cystogram to have a contrast extravasation from the posterior-dependent portion of the bladder and a large retrovesical fluid collection. On exploratory laparotomy, a leak from the posterior portion of the bladder was confirmed. Owing to the conditions in the abdomen and the patient's obese body habitus, the perforation was very difficult to view. A 17F rigid cystoscope was utilized and the perforation was identified on the posterior inferior portion of the bladder at the anastomotic line. A wire was passed through the perforation into the abdomen where it was seen and an 18F council catheter was then placed in an antegrade manner from the abdomen. Placement of the catheter and inflation of the balloon did not cause any additional apparent damage to the bladder mucosa. With the catheter on traction, the dependent bladder could be pulled back into the operative field, allowing complete observation of the defect for water-tight two-layer closure.

***Conclusion:*** Bladder perforation after augmentation cystoplasty is a potentially life-threatening complication that can be difficult to repair. This article serves to present a novel way to identify and facilitate repair of the defect intraoperatively using endourologic principles for a posterior defect.

## Introduction and Background

Augmentation cystoplasty for the management of neurogenic bladder has been performed with increasing frequency since 1967 when Servadio first reported its use in a patient with sacral agenesis and neuropathic incontinence.^1^ This procedure, although effective to treat the small-capacity high-pressure bladder, has multiple well-known complications. The most dangerous of these complications is bladder perforation, which has a mortality rate of 23% to 25% in large part caused by delayed presentation and sepsis.^2–4^ At the time of presentation, these patients often have a hostile acute abdomen, extensive adhesive disease, and loculating urinomas. Because of these factors, identification of the site of perforation can be extremely challenging. This article discusses a novel method for identifying the perforation using endourologic techniques to allow for easier repair.

## Presentation of Case

A 24-year-old woman with a history of spina bifida presented to the emergency department with a 2-day history of decreased oral intake, nausea, vomiting, fevers, diffuse abdominal pain, and distention. She initially underwent ileal augmentation cystoplasty with appendicovesicostomy and rectus fascia bladder neck sling 5 years ago and was managed with intermittent catheterization every 3 to 4 hours. On detailed history, it was found that the patient had been failing to wake in the early morning to catheterize herself with durations between catheterization of up to 10 to 12 hours. Based on the history taken from the family, it was apparent that the patient had not been compliant with the prescribed catheterization regimen.

**FIG. 1.**
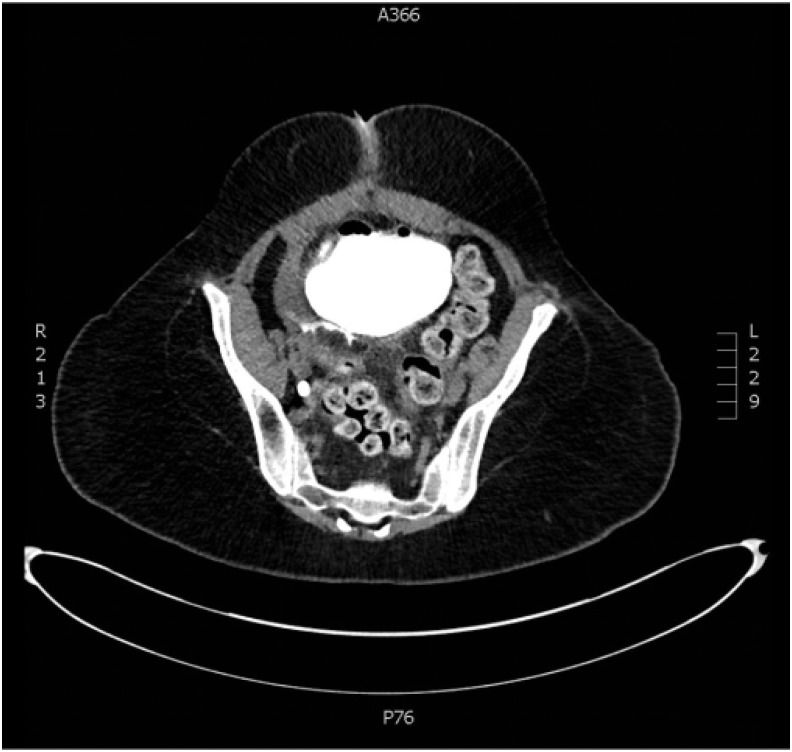
Axial imaging of CT cystogram showing posterior perforation with extravasation.

**FIG. 2.**
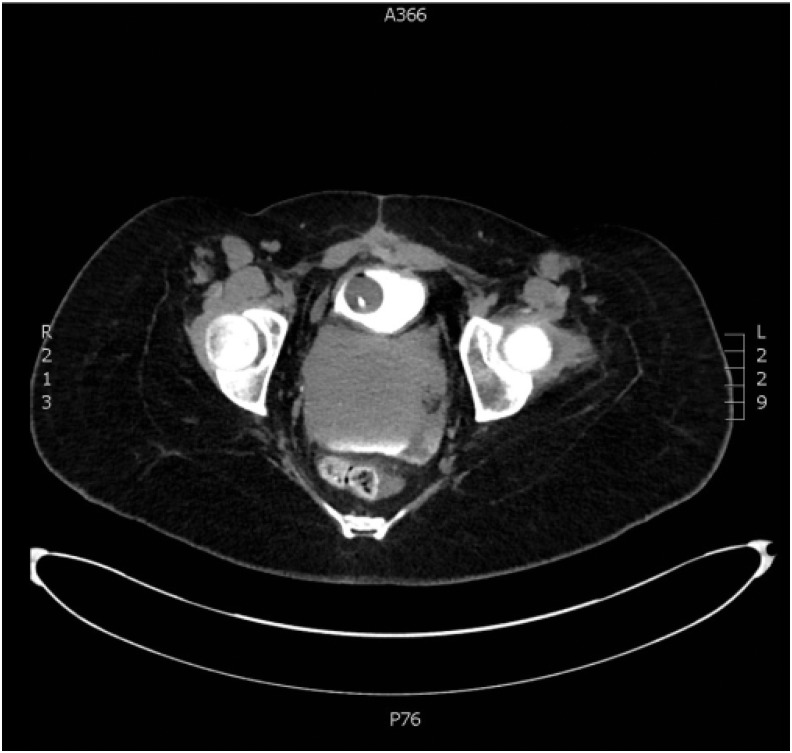
Axial imaging of CT cystogram showing posterior fluid collection.

**FIG. 3.**
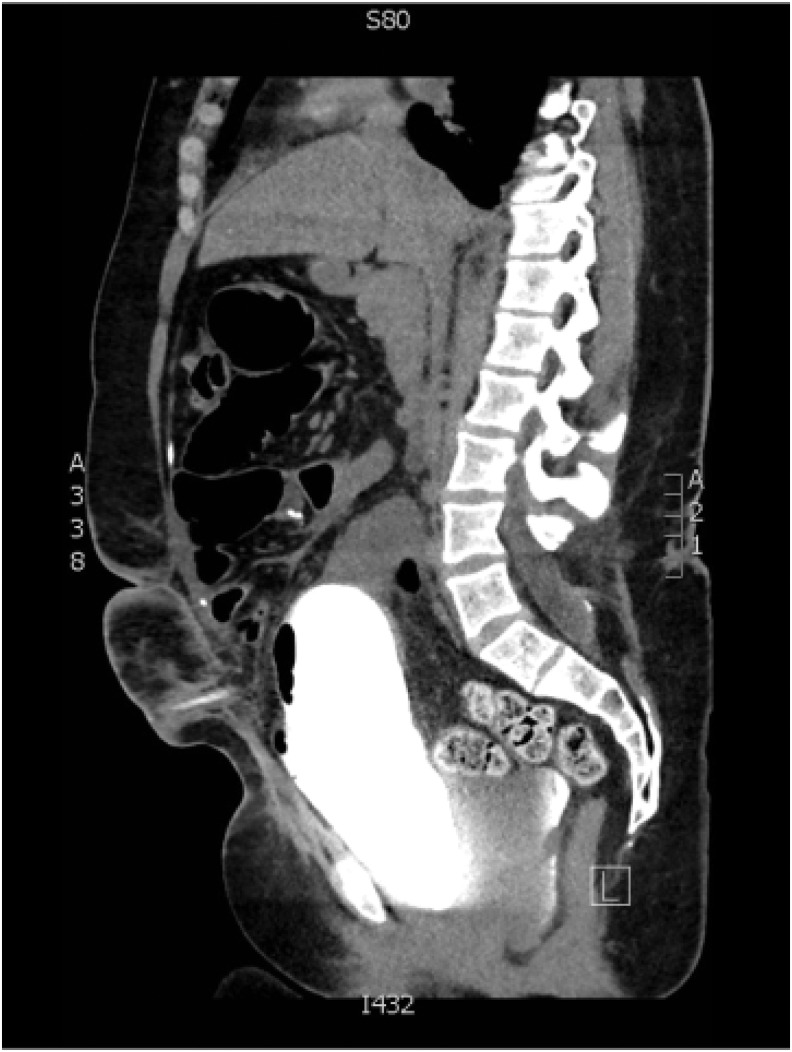
Sagittal imaging of CT cystogram showing posterior fluid collection and extravasation.

On examination, she was febrile and had diffuse abdominal tenderness and distention. She had a leukocytosis and CT of abdomen and pelvis with intravenous contrast revealed dilated loops of bowel with free fluid in the pelvis and concern for a transition point suggestive of small bowel obstruction. Given her history of bladder reconstruction and free fluid on imaging, urology was consulted and a CT cystogram was obtained. The cystogram showed contrast extravasation from the posterior-dependent portion of the bladder in association with a large retrovesical fluid collection (Figs. 1–3). The patient was taken immediately to the operating room for exploratory laparotomy with general surgery and urology.

At laparotomy, murky fluid filled the abdomen, yet no bowel obstruction or perforation was encountered. With catheters in the bladder and stoma, adhesiolysis was performed to identify the appendicovesicostomy and the bladder. The bladder was filled with methylene blue, confirming a leak from the posterior portion of the bladder. Owing to the conditions in the abdomen and the patient's obese body habitus, the perforation was very difficult to view. Transvesical approach through the augmented segment was an option for repair, but rather than potentially causing vascular compromise of the augment limb, we decided on cystoscopy to help with identification of the perforation. A 17F rigid cystoscope was passed per urethra and the perforation was identified on the posterior inferior portion of the bladder at the anastomotic line. A hybrid guidewire was passed through the perforation into the abdomen where it was easily seen and secured. An 18F council catheter was then placed in an antegrade manner from the abdomen into the bladder. With the catheter balloon on traction, the dependent bladder could be pulled back into the operative field, allowing complete observation of the defect for water-tight two-layer closure.

The patient's recovery was complicated by a persistent ileus; however, she was discharged to home on postoperative day 11 after a course of IV antibiotics. Her CT cystogram on postoperative day 21 revealed an intact repair and a pseudocyst associated with her ventriculoperitoneal shunt requiring externalization. She has resumed clean intermittent catheterization every 4 hours and has continued to do well.

## Discussion

This case illustrates many of the challenges in identifying and managing bladder perforation after augmentation. The presentation of these cases can vary from nausea with vomiting, diffuse abdominal pain to shoulder pain from peritonitis.^3^ It is imperative that clinicians have a high index of suspicion and treat abdominal pain with distention, nausea, vomiting, or fever in a patient with history of augmentation as a perforation until proven otherwise. This is especially important given the historical unreliability of the plain film cystogram in patients who have undergone augment given the redundancy of the bowel and the occasional increased capacity. There are important technical considerations that include adequate filling and multiple views when using plain film to evaluate the redundant contours. In addition to this, postdrainage films must be obtained.^4^ Despite correct technique, there is still a high false negative rate, historically ranging from 9% to 57%^2^ furthering a delay in diagnosis, and technical intricacies may necessitate involvement of an urologist.

There are several reports of patients who have been effectively managed nonoperatively^2,3^; however, the standard of care remains exploratory laparotomy with identification and repair of the defect, large volume irrigation, and drain placement. Opening the augmented bladder to inspect from the inside out is an alternative approach in cases where the defect cannot be readily identified but carries the risk of impaired healing from ischemia. It has been shown that the bowel segments used for augmentation have areas of ischemia necrosis presumably from periods of high pressure.^4^ This can not only cause perforation at the area of weakness but can also weaken a closure if the bladder is opened through the bowel segment.

Postoperative management includes catheter drainage for 2 to 4 weeks with cystogram at the time of catheter removal that includes postdrainage films at multiple angles. These patients have been shown to be at risk for recurrence of their perforation without behavioral modifications. Risk factors for perforation have been examined and behavioral factors have been implicated, with the highest level of concern in patients with substance abuse and other mental disorders.^3^ It is, therefore, imperative that these patients receive regular counseling and follow-up for reiteration of the risks associated with catheter noncompliance.

## Conclusion

Bladder perforation after augmentation cystoplasty is a potentially life-threatening complication that can be difficult to repair. This article serves to present a novel way to identify and facilitate repair of the defect intraoperatively using endourologic principles for a posterior perforation. Utilization of endoscopic techniques to not only observe the defect but also to bring the bladder perforation into the field of view allows for an easier repair to be performed without the risk of compromised blood supply to the bowel segment utilized. In addition to this, continued work must be done regarding counseling and follow-up with these patients to reduce the risk of recurrent perforation by mitigating modifiable risk factors associated with perforation.

## Abbreviation Used

CT: computed tomography
